# Left Atrial Myxoma as a Rare Cause of Acute Coronary Syndrome: A Case Report

**DOI:** 10.3390/reports9010062

**Published:** 2026-02-13

**Authors:** Ismail Mousati, Paul Vermeersch, Nathalie Meyten

**Affiliations:** HartCentrum, Ziekenhuis Aan de Stroom (ZAS) Middelheim, Lindendreef 1, 2020 Antwerp, Belgium

**Keywords:** acute coronary syndrome, cardiac tumor, myxoma, transesophageal echocardiography

## Abstract

**Background and Clinical Significance**: Atrial myxomas are rare, benign cardiac tumors, most commonly originating in the left atrium, with potential for serious embolic and obstructive complications. **Case Presentation**: We report a 67-year-old woman presenting with acute chest pain and NSTEMI whose coronary angiography results were normal. Echocardiography revealed a large, heterogeneous, stalk-attached left atrial mass, confirmed by transesophageal imaging. Surgical resection was performed two days after diagnosis, and histopathology confirmed it was an atrial myxoma. Postoperative recovery was uneventful, with reassuring follow-up echocardiography. **Conclusions**: Echocardiography remains an invaluable tool for the identification of atrial myxomas and early surgical excision is critical, as they have an excellent prognosis.

## 1. Introduction and Clinical Significance

Cardiac myxomas are rare, benign primary cardiac tumors, yet they are of substantial clinical importance due to their potential to cause serious and life-threatening complications [1,2].

Although histologically benign, myxomas can lead to systemic embolization, intracardiac obstruction, and constitutional symptoms, resulting in diverse clinical manifestations such as ischemic stroke, syncope, heart failure, and, more rarely, acute coronary syndrome (ACS) [1,2,3].

Atrial myxomas are the most common primary cardiac tumors in adults, accounting for approximately half of all cases, and most frequently arise from the left atrium, typically originating from the interatrial septum near the fossa ovalis [3]. They most often present in middle-aged adults and show a female predominance [4]. While embolic events are well-recognized complications, coronary artery embolization leading to ACS remains an exceptionally rare presentation due to the protective anatomy of the coronary ostia and the typically larger size of embolic fragments [4].

Echocardiography plays a central role in the evaluation of suspected intracardiac masses and is the cornerstone of diagnosis for cardiac myxomas. It enables accurate assessment of tumor morphology, size, mobility, and attachment site, as well as the identification of associated hemodynamic consequences, including valvular obstruction or regurgitation [5]. Transesophageal echocardiography offers superior spatial resolution and is particularly valuable in cases where transthoracic imaging is inconclusive or when detailed anatomical characterization is required [3,4,5].

The recognition of atrial myxoma as an uncommon etiology of ACS is clinically significant, as the corresponding management strategies differ substantially from those for atherosclerotic coronary disease. Failure to identify the underlying cardiac source may result in recurrent embolic events with potentially fatal consequences. This case report highlights atrial myxoma as a rare cause of ACS and underscores the importance of comprehensive cardiac evaluation, including echocardiography, in patients presenting with myocardial infarction in the absence of traditional cardiovascular risk factors or angiographic evidence of coronary atherosclerosis.

## 2. Case Presentation

**Figure 1:**
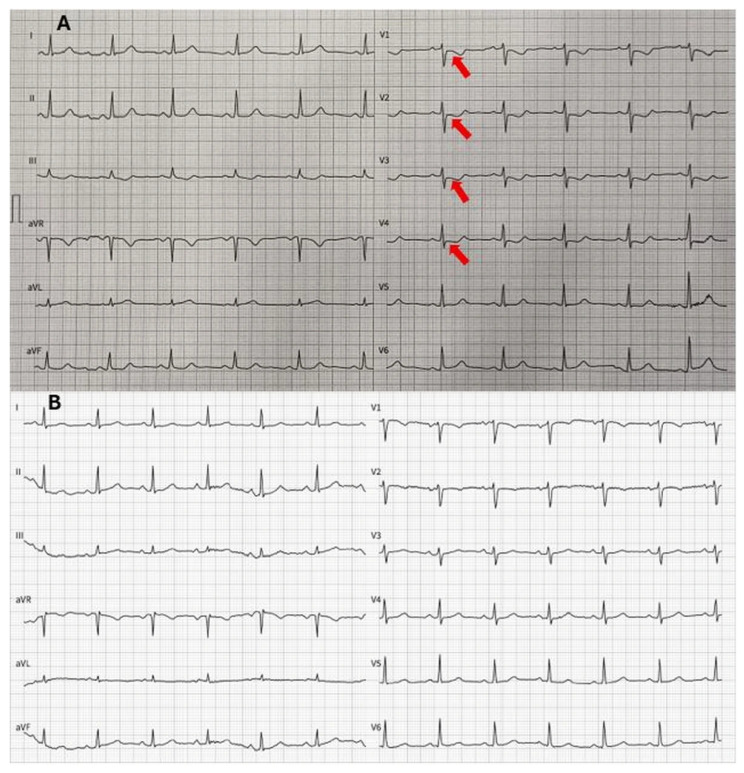
**Electrocardiogram (ECG).** (**A**) Before surgical resection of left atrial myxoma. Arrows indicate mild ST depression in the anteroseptal leads. (**B**) Two days after surgical resection of left atrial myxoma. Resolution of the repolarization abnormalities in the anteroseptal leads.

A 67-year-old woman presented to the emergency department with acute-onset chest pain. She had no significant medical history and no known cardiovascular risk factors. On initial evaluation, the patient was hemodynamically stable, and physical examination revealed no cardiac murmurs or signs of heart failure. The admission electrocardiogram (ECG) revealed mild anteroseptal ST-segment depression, raising concern for myocardial ischemia (Figure 1).

Initial laboratory testing showed an elevated high-sensitivity cardiac troponin level of 2.3 ng/L, which increased to 23.8 ng/L on repeat measurement one hour later, consistent with ongoing myocardial injury. Based on the clinical presentation, ECG findings, and dynamic troponin rise, a diagnosis of non-ST-segment elevation myocardial infarction (NSTEMI) was made. The patient was treated according to the standard NSTEMI protocol, and semi-urgent coronary angiography was performed to scan for coronary artery disease.

**Figure 2:**
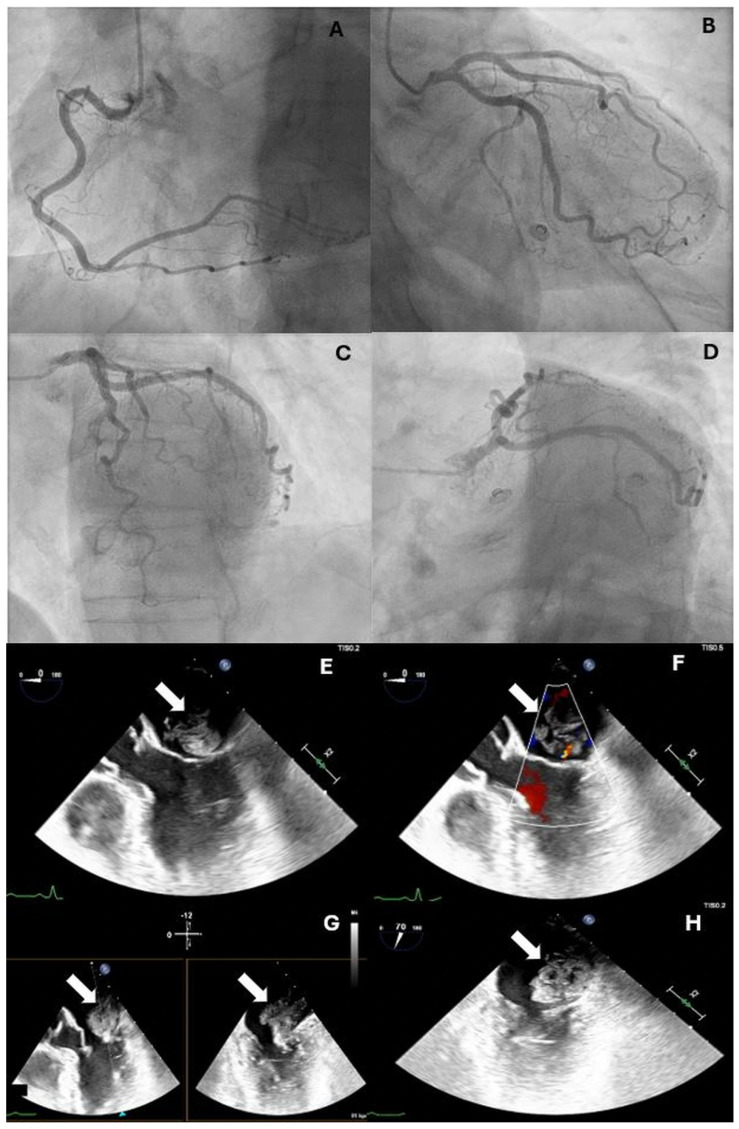
Coronary angiography and transesophageal echocardiography of left atrial myxoma. (**A**) Normal right coronary artery (RCA). (**B**–**D**) Normal left main stem (LMS), left anterior descending artery (LAD), and left circumflex branch (LCx). (**E**–**H**) Large mass with an irregular, heterogeneous appearance. The image reveals that it is attached by a stalk to the lateral wall of the left atrium. There is no evidence of vascularization.

Coronary angiography revealed normal coronary arteries without evidence of atherosclerotic disease, thrombosis, or dissection (Figure 2, Videos S1–S3), prompting further investigation into an alternative etiology for the myocardial infarction. Subsequent transthoracic echocardiography revealed a mobile, protruding mass within the left atrium. It appeared to originate from the lateral wall. Moreover, there were no regional wall motion abnormalities. To further characterize the lesion, transesophageal echocardiography was performed, which revealed a heterogeneous, irregularly shaped mass measuring approximately 3 cm × 2 cm. The mass was attached to the lateral wall of the left atrium by a stalk and showed no evidence of internal vascularization, findings highly suggestive of an atrial myxoma (Figure 2, Videos S4–S8).

Given the risk of systemic embolization, including cerebral events, a cerebral computed tomography scan was conducted to exclude acute ischemic stroke; the results were reassuring. After multidisciplinary discussion, the patient was referred for semi-urgent surgical intervention. Surgical excision of the left atrial mass was performed two days after diagnosis without complications.

Histopathological examination of the resected specimen revealed fragments originating from the atrial wall, predominantly composed of muscle fibers. The tumoral mass consisted of myxoid stroma and neoplastic cells. The cells were polygonal with lightly eosinophilic cytoplasm and indistinct cell borders. The nuclei were ovoid and displayed an open chromatin pattern, with inconspicuous nucleoli. Focal inflammatory cells and hemosiderin deposition were present within the stroma. There was no evidence of necrosis or mitotic activity. These histopathological findings confirmed the diagnosis of an atrial myxoma. The postoperative course was uneventful, and the patient was extubated shortly after surgery. She was transferred from the intensive care unit to the general ward on postoperative day one. Follow-up ECG after surgery revealed normalization of the ST depression (Figure 1). Follow-up transthoracic echocardiography performed three days after surgery revealed normal cardiac function without residual intracardiac mass or valvular abnormalities.

After a total hospital stay of nine days from initial presentation, the patient was discharged in good clinical condition, and she was completely asymptomatic at the time of discharge.

## 3. Discussion

Primary cardiac tumors are exceedingly rare, with myxomas representing the most common type [3,5]. Advances in multimodality imaging have led to more frequent antemortem diagnosis of cardiac masses in recent years [6]. The classic symptom triad includes systemic embolic events; intracardiac obstructive manifestations such as dyspnea upon exertion, pulmonary edema, and right-sided heart failure; and constitutional symptoms, including fever, weight loss, and myalgia [5,6].

Our patient presented with a non-ST-segment elevation myocardial infarction caused by coronary artery embolization from a left atrial myxoma. ECG abnormalities, including those resembling ischemia (ST-segment deviation, both depression and elevation, and a negative T-wave), are prevalent in cardiac tumors [7]. After surgery, the ST-segment depression resolved in our patient.

Upon physical examination, a diastolic murmur over the mitral area is commonly audible due to left ventricular inflow obstruction, and a late diastolic tumor sound may be heard after the second heart sound when the mass prolapses into the left ventricle. In this case, only the late diastolic murmur was heard since there was no left ventricular inflow obstruction.

Transthoracic echocardiography is still the primary diagnostic modality, and it is an easy accessible diagnostic tool. Transesophageal echocardiography offers higher sensitivity and provides detailed information regarding the site of attachment, mobility, involvement of other cardiac chambers, and characteristic gross pathological features such as calcification, necrosis, and intratumoral hemorrhage. Multimodality imaging, including computed tomography (CT) and cardiac magnetic resonance (CMR), may further aid in assessing extracardiac extension and tissue characterization [5,8]. In this case, the absence of CMR represents a limitation, as the diagnosis of MINOCA cannot be definitively confirmed without tissue characterization. Another differential diagnosis to consider in this context is type 2 myocardial infarction. In this case, inflammatory markers were assessed and did not suggest a systemic inflammatory or infectious process. Hemodynamic parameters remained stable throughout hospitalization, and there was no evidence of severe anemia, hypoxia, or sepsis.

Surgical excision is the treatment of choice for atrial myxomas and should be performed as early as possible following diagnosis, particularly for symptomatic patients [1,2,3]. Complete resection is associated with an excellent prognosis and a low risk of recurrence [5,8].

## 4. Conclusions

Atrial myxomas may present with symptoms; however, they are often asymptomatic and often diagnosed incidentally during imaging. Echocardiography remains an invaluable tool for the early identification of intracardiac masses such as atrial myxomas, making it critical for preventing embolic events. The treatment of choice for atrial myxomas is surgical excision. The recommended timing of surgery is as early as possible after diagnosis, especially if the tumor is symptomatic. The prognosis is excellent after complete resection, with low recurrence rates.

## Data Availability

All relevant data are included within the article. Additional de-identified information can be made available from the corresponding author upon reasonable request, in accordance with patient confidentiality requirements.

## Supplementary Materials

The following supporting information can be downloaded at https://www.mdpi.com/article/10.3390/reports9010062/s1. **Video S1. Coronary angiography of the right coronary artery:** Normal right coronary artery. **Videos S2 and S3. Coronary angiography of the left coronary arteries:** Normal left main stem (LMS), left anterior descending artery (LAD) and left circumflex branch (LCx). **Videos S4, S6 and S8. Transesophageal echocardiography of left atrial myxoma:** Large mass in the left atrium with an irregular, heterogeneous appearance and attached by a stalk to the lateral wall of the left atrium and protruding towards the left ventricle in systole. **Videos S5 and S7. Transesophageal echocardiography of left atrial myxoma:** No signs of vascularization in the mass and associated mild mitral valve regurgitation.

## List of Abbreviations

CMR	Cardiac magnetic resonance
CT	Computed tomography
ECG	Electrocardiogram
LAD	Left anterior descending
LCx	Left circumflex
LMS	Left main stem
NSTEMI	Non-ST-elevation myocardial infarction
